# Miniaturized Ceramic-Based Microbial Fuel Cell for Efficient Power Generation From Urine and Stack Development

**DOI:** 10.3389/fenrg.2018.00084

**Published:** 2018-10-01

**Authors:** Iwona Gajda, Andrew Stinchcombe, Irene Merino-Jimenez, Grzegorz Pasternak, Daniel Sanchez-Herranz, John Greenman, Ioannis A. Ieropoulos

**Affiliations:** 1 Bristol Robotics Laboratory, Bristol BioEnergy Centre, University of the West of England, Bristol, United Kingdom; 2 Department of Applied Sciences, University of the West of England, Bristol, United Kingdom

**Keywords:** bioenergy, microbial fuel cell, urine, ceramic membrane, stacking, usable power, module

## Abstract

One of the challenges in Microbial Fuel Cell (MFC) technology is the improvement of the power output and the lowering of the cost required to scale up the system to reach usable energy levels for real life applications. This can be achieved by stacking multiple MFC units in modules and using cost effective ceramic as a membrane/chassis for the reactor architecture. The main aim of this work is to increase the power output efficiency of the ceramic based MFCs by compacting the design and exploring the ceramic support as the building block for small scale modular multi-unit systems. The comparison of the power output showed that the small reactors outperform the large MFCs by improving the power density reaching up to 20.4 W/m^3^ (mean value) and 25.7 W/m^3^ (maximum). This can be related to the increased surface-area-to-volume ratio of the ceramic membrane and a decreased electrode distance. The power performance was also influenced by the type and thickness of the ceramic separator as well as the total surface area of the anode electrode. The study showed that the larger anode electrode area gives an increased power output. The miniaturized design implemented in 560-units MFC stack showed an output up to 245 mW of power and increased power density. Such strategy would allow to utilize the energy locked in urine more efficiently, making MFCs more applicable in industrial and municipal wastewater treatment facilities, and scale-up-ready for real world implementation.

## INTRODUCTION

Observing the continuously increasing demand for water and energy in the world, alternative sources are needed to meet the requirement of a growing population. Microbial Fuel Cell (MFC) represents one sustainable technology that directly converts organic biomass contained in wastewater into electric current. Thus, it can be a potential alternative source for energy and water clean-up (Habermann and Pommer, 1991; Pant et al., 2010). Microbial Fuel Cells generate electric current as a direct result of microbial metabolism, where the anodic biofilm is the engine of the process, utilizing substrates, and converting chemical energy to electrical energy. MFC technology development into commercial applications has been limited by the high cost of materials and the low efficiency of the energy recovered. Therefore, the successful scale-up process should involve the optimization of materials and design which allow a cost and energy effective technology (Do et al., 2018), as well as more lab-based and field trial led research for development of this technology for large scale applications (Khan et al., 2017). With this approach in mind, recent advancements bring the technology closer to the real life implementation thanks to using ceramic for MFC architecture (Gajda et al., 2015a,c; Pasternak et al., 2016), open to air cathodes with non-platinum catalysts (Merino-Jimenez et al., 2016; Gajda et al., 2018) and improved design of multiple MFC units in the system (Ieropoulos et al., 2008). These advancements were recently presented in the Pee Power^R^ stack including multiple MFC units divided in modules. Each module contained several MFCs connected both fluidically and electrically in parallel, whilst the modules could be connected in an electrical array both in series and in parallel, and fluidically connected in a cascade configuration. The Pee Power^R^ stack was tested in the field demonstrating its capability to be used in real life applications by powering the internal lighting of a urinal, only from the urine provided by the users (Ieropoulos et al., 2016). However, the power output achieved during this trial should be improved (Ieropoulos et al., 2016). Modular approach is being developed in numerous studies (Kim et al., 2010; Dong et al., 2015; Ge and He, 2016) and it shows a good strategy for the implementation in wastewater treatment facilities (Liang et al., 2018). However, low performance in large-scale systems is almost always linked to transport limitations, including ionic transport and acidity/alkalinity transport from the electrodes (Popat and Torres, 2016). Efficiency improvement could be achieved by the miniaturization, as previously shown, where smaller units outperformed larger reactors of equivalent volume and geometric foot-print (Ieropoulos et al., 2010); also by increasing the surface area-to-volume ratio and decreasing the distance between the electrodes (Qian and Morse, 2011). For example, a micro-scale, flat-plate MFC with a high surface area to volume ratio enhances mass transfer coefficient leading to improved power density (Ren et al., 2014). In addition, the integration of an improved design in stacked modules results in a more efficient electrochemical treatment and higher usable electricity levels, for example to power indoor lighting. However, the power output obtained in small scale reactors does not linearly increase in proportion with the scale of the reactor (Logan et al., 2015). Thus the power density, which represents the actual power of the MFC divided by reactor volume (W/m^3^) or the surface projected area of the electrode (W/m^2^), generally decreases with increasing scale. The changes in power density during scale-up result from changes in many important factors, such as reactor volume, electrode spacing and electrode specific surface area (surface area per volume; Cusick et al., 2011) which determine internal resistance of the system (Ieropoulos et al., 2008).

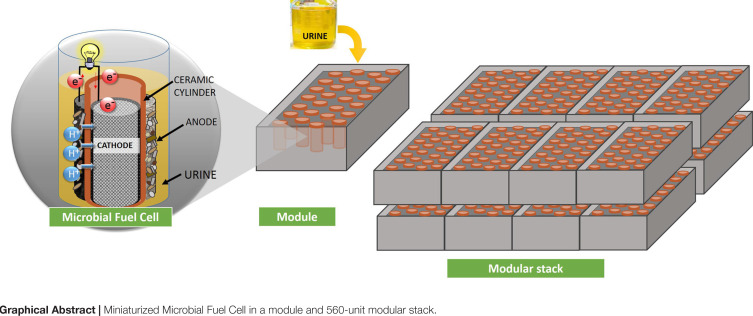



Smaller MFC reactors benefit from lower activation losses, higher substrate utilization (mass transfer) and improved diffusion of protons H^+^ out of the biofilm (Torres et al., 2008). Furthermore, by increasing the effective surface area while maintaining short proton diffusion lengths results in higher surface area-to-volume (SAV) ratio and a more efficient use of substrates per unit volume (Wang et al., 2011; Walter et al., 2016).

MFCs as power sources for environmental sensors is nearing practical use as it offers electricity generation without a need for recharging (Pasternak et al., 2017), showing the stability of the anodic biofilm (You et al., 2015) with a continuous power production. Utilizing urine and wastewater as an energy source has become one of the major research routes toward not solely energy harvesting but also nutrient recovery (Kuntke et al., 2014), production of clean catholyte (Merino Jimenez et al., 2017) and has been shown as a practical demonstration in real life scenarios and a showcase of the MFC technology to the wider public (Ieropoulos et al., 2016).

In this work we look into the ceramic reactor size, where ceramic architecture is important since the ceramic material is also acting as a membrane. The aim is to increase power production from a decreased footprint of the whole system and to identify the optimum design and running conditions for large scale manufacturing and operation of MFC stacks. As part of this aim we present for the first time the development of a stack made of 560 miniaturized MFC units to study power production from urine in laboratory conditions. This work presents a technology scale-up through the miniaturization and multiplication of the individual components resulting in the overall decrease in the total footprint of the final modular stack. The presented design does not use mediators, buffers, chemical catalysts, or cation exchange membranes therefore it is focusing on the practical approach of the modularly built MFC systems for the implementation in real world conditions.

## MATERIALS AND METHODS

### Individual MFCs

#### Electrodes

**FIGURE 1:**
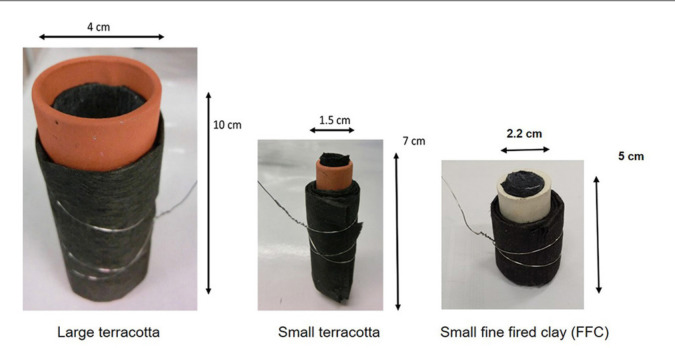
Individual MFCs tested using large and small terracotta cylinders.

Anodes were made of carbon veil fiber (20 g/m^2^, PRF Composites, UK) cut to the desired size (**Table 1**), folded and wrapped around the ceramic cylinder attaching a stainless steel wire (dia. 0.5 mm) as a current collector (**Figure 1**). The cathodes were prepared in house by mixing Activated Carbon powder (G Baldwins and Co., UK) and 20% PTFE (60% dispersion in H_2_O, Sigma Aldrich, UK) applied onto PTFE treated carbon veil sheet as previously described (Gajda et al., 2015a). The cathode material was cut according to the required dimensions (**Table 1**) and placed inside the ceramic cylinder as shown in **Figure 1**. The anode to cathode ratio was evaluated by keeping it at ∼27 (anode total geometric size was 27 times larger than the cathode) and comparing it to the ratio of 14 (**Table 1**).

**TABLE 1:** Parameters of the MFCs used in this study.

Name	Ceramic cylinder	Height (cm)	Diameter (cm)	SAV of the ceramic (cm^−1^)	Anode area (cm^2^)	Cathode area (cm^2^)	Anode to cathode area ratio
	
LargeT	Large terracotta	10	4	1	2,430	90	27
SmallT	Small terracotta	7	1.5	2.7	560	21	27
S FFC	Small fine fired clay	5	2.2	1.8	560	20	28
S FFC 14	Small fine fired clay	5	2.2	1.8	280	20	14

#### Small Terracotta MFCs

Terracotta MFCs were made out of small scale terracotta cylinders which were hand made out of fired, neutral clay and open at both ends (70 mm long, 15 mm diameter, 2 mm thickness) (Aquaforest, Ireland). One end was sealed with a plastic stopper and non-toxic sealant (Wet Water Sticky Stuff, Aquatrix, UK).

The other two types of small scale ceramic MFCs used in this study were named S FFC and S FFC 14 assembled using fine fire clay (FFC) cylinders (ROCA, Spain). The cylinders dimensions were 50 mm height, 22 mm outer diameter and 3 mm thickness. They were assembled in two configurations where the anode/cathode ratio was 28 and 14, respectively. All MFCs were tested in triplicates.

#### Large Ceramic Reactors

The MFCs were compared against large tubular MFCs made of terracotta (Weston Mill Pottery, UK) (100 mm long, 42 mm diameter, 3 mm thickness) with the same anode to cathode ratio (27:1) as the small reactors and operated in laboratory conditions.

The difference in thickness is a parameter that was outside of our control, in acquiring these cylinders and setting them up as MFC units. All other parameters were kept (proportionately) the same.

### Modular Boxes

**FIGURE 2:**
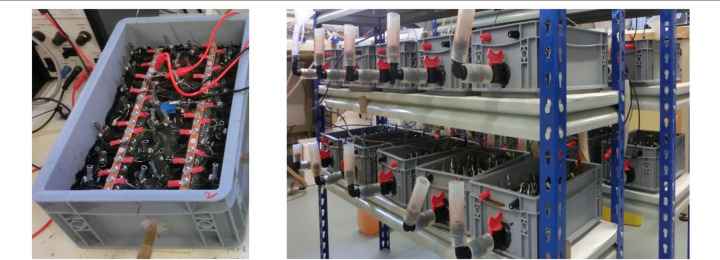
A single small scale module consisting of 28 MFC units (S FFC 14) and a modular stack configuration made of 20 modules (560 MFCs in total).

The large modular box was assembled as previously described (Ieropoulos et al., 2016). The small modular box consisted of a 5 L plastic container (Plastor, UK) used as the chassis with an inlet and an outlet to allow the urine flow (**Figure 2**). The S FFC ceramic cylinders (**Table 1**) were chosen to use for the assembly due to the availability of this product in large numbers. Anodes and cathodes were the same as in the individual S FFC 14 setup (**Figure 1**) with the anode to cathode ratio of 14. Again, this is due to the large number of units required for the construction of the module and this ratio was chosen in order to reduce the cost of the anode electrode material used for the whole stack. Once the 28 MFC units were installed in the plastic container, all the anodes and the cathodes were connected in parallel electrical configuration (**Figure 2**).The total volume of the box was 1.8 L and it was inoculated with a 1:1 activated sludge and urine mix and kept in batch mode using neat human urine as a feedstock which was replenished daily.

A modular stack was assembled using 20 modules making a total of 560 MFC units which again were identical to S FFC 14 used in the individual tests. The boxes were fluidically connected using L-shaped and T-shaped connectors and silicone tubing allowing air gaps between each individual module. Electrically paired MFC modules were connected in parallel resulting in the total of 10 pairs which were then connected in series. Due to large quantity of urine required for the stack operation, artificial urine were prepared (**Table S1**) and mixed with real human urine in the 75% : 25% ratio. The modules were arranged in two fluidic cascades and periodically fed with the prepared mixture.

### Operation and Data Monitoring

All individual MFC units were placed in plastic containers of 200 mL for Large MFCs and 50 mL volume for small MFCs. The MFCs were inoculated with anaerobic activated sewage sludge (Wessex Water, UK) of neutral pH and periodically fed with neat human urine (pH 9–9.4) in batch mode to evaluate the rate of performance. The MFC reactors were tested in triplicates under 100 Ω resistor. Polarization curve experiments were performed using an automated device applying a range of resistances from 30 k Ohm to 3 Ohm in the time intervals of 3 min (Degrenne et al., 2012). For the 560-unit modular stack, the polarization curve experiments were performed using a variable resistance decade box and changing resistances every 30 min within the range of 500–0.5 Ohm.

## RESULTS AND DISCUSSION

**FIGURE 3:**
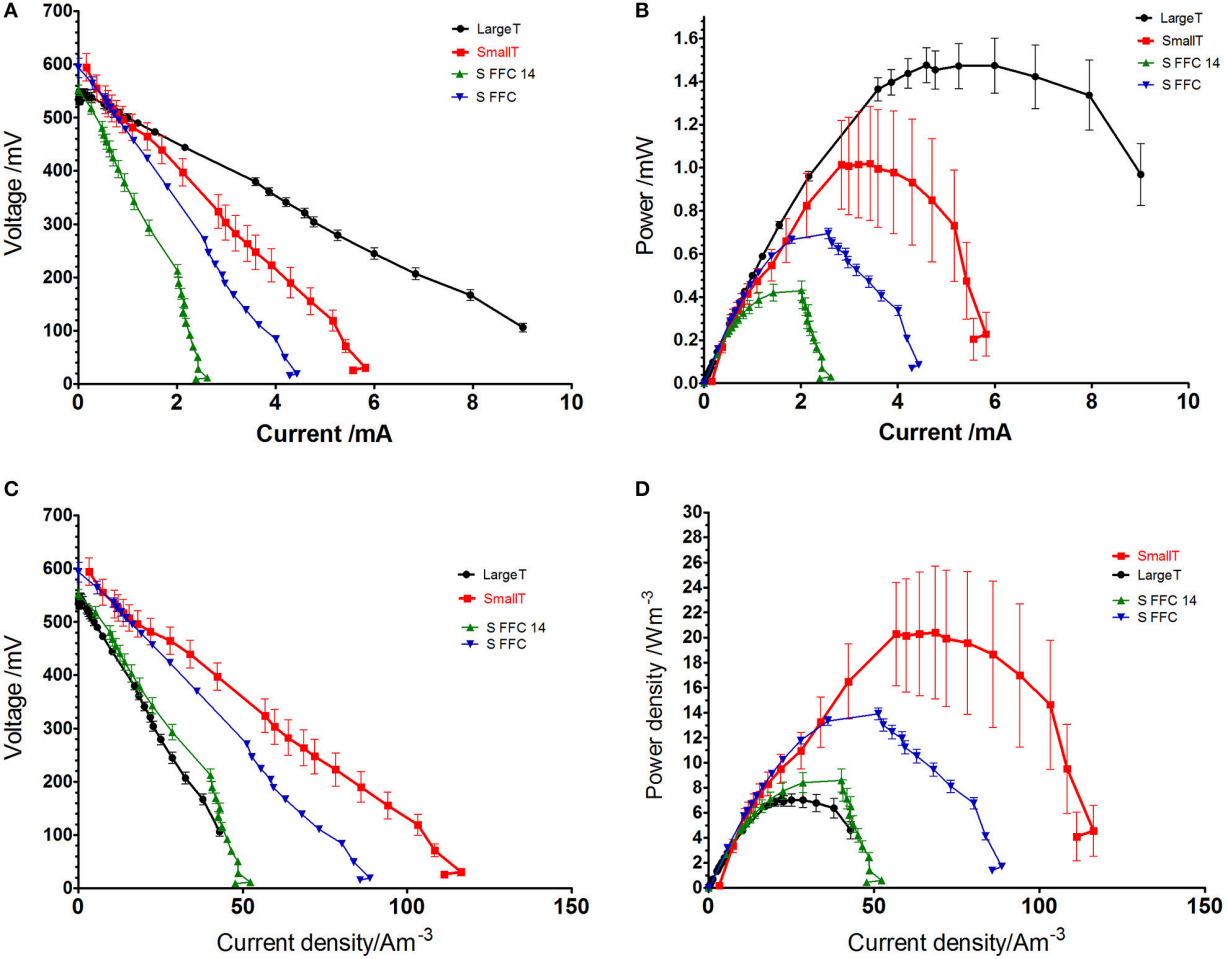
Polarization data showing actual (A) and current density values (C) when the individual cylinders are compared. Power curve data showing actual (B) and power density (D) values.

In terms of absolute power a single large terracotta MFC (LargeT) produced a maximum of 1.47 mW, while a small terracotta (SmallT) unit produced 1.01 mW (**Figure 3B**). The small reactors made of fine fired clay (S FFC14) generated 0.43 mW and 0.69 mW when the anode surface area was doubled (S FFC) to keep the same anode to cathode area ratio used in LargeT and SmallT (**Table 1**). The maximum performance (**Figure 3D**) achieved during polarization experiment showed that volumetric density of the SmallT was 20.4 W/m^3^ (mean value) while LargeT units achieved only 7.0 W/m^3^ which suggests 2.9 times higher performance of small MFCs. This supports the fact that smaller MFC devices can take advantage of high surface to volume ratio, which in this case was calculated as 2.67 cm^−1^ for SmallT, 1.81 cm ^−1^ for FFC and 1.0 cm ^−1^ for LargeT units (**Table 1**). Fine fired clay performance was lower than terracotta and it might be due to the type, thickness, porosity and the SAV of the ceramics used. The higher SAV of the reactor, the better fuel mass transfer achieved and the smaller resistance obtained (Tsai et al., 2017).

Results showed that the small scale MFCs benefit from improved power density performance in comparison to the large tubular MFCs. The large error bars in the electrochemical behavior of the triplicate in the terracotta MFC was replicable in repeated polarization data however the diversion of the performance persisted. This might be due to small diameter of the cathode chamber, which is not easily accessible and it does not allow a stable contact, also the connection of the stainless steel crocodile clip onto the cathode is difficult. As the cathodes were prepared manually and the stability of the electrical connection to the cathode in a narrow cathode chamber remains a challenge.

However, more research is necessary to fully characterize and large deviation even within the same experimental group.

**FIGURE 4:**
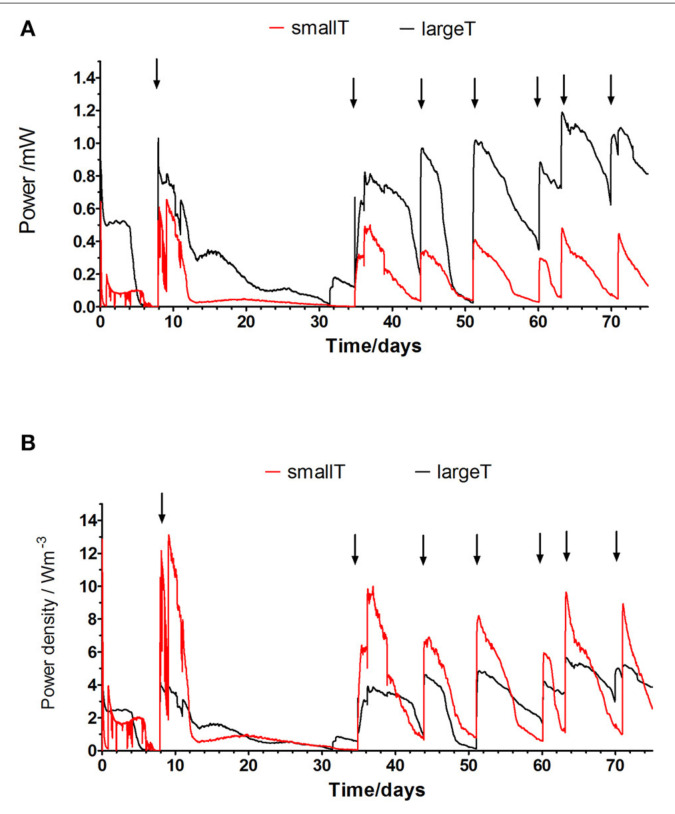
(A) Absolute power of the SmallT and LargeT MFCs tested (mean value of triplicated data) using urine as a feedstock. (B) Power density of the individual SmallT and LargeT units. Arrows indicate the addition of new feedstock (urine).

The comparison between the two anode/cathode ratios used in FFC configurations suggest that the larger anode electrode area gives a 60% higher power output. It might be due to the increased total surface area and the anode packing into more three-dimensional structure for allowing biofilm growth and sufficiently large channels for substrate supply and product removal (Baudler et al., 2014). For the future construction of the MFC stacks, the SAV of the ceramic material should be taken into account as well as its type, composition, thickness and porosity. This work might indicate that the increased SAV of the ceramic structures used here as membranes is favorable and it also determines the size of the whole reactor. **Figure 4** shows the temporal behavior of the large and small terracotta cylinders. The maximum power output was 1.15 mW achieved by the LargeT MFCs and 0.63 mW for the small reactors and in the power density terms that shows up to 12.7 W/m^3^ generated by the small reactors and 5.3 W/m^3^ for the large MFCs. The data indicates that the small scale MFCs require frequent feeding as the power generation drops more rapidly than in the case of large MFCs. This might suggest quicker utilization of available feedstock and implies using faster flow/rate/ frequent feeding regime for the miniaturized MFC systems.

During electrochemical operation, the production of catholyte on the surface of the cathode electrode was also observed and driven by electric current. The accumulation of catholyte is primarily driven by electroosmotic drag, therefore the current produced by the MFC (Kim et al., 2009; Gajda et al., 2015b) and it the allows collection of basic catholyte in the ceramic inner cathode chamber (Gajda et al., 2015a; Merino Jimenez et al., 2017). It shows a potential of simultaneous treatment of urine by ion separation (Kuntke et al., 2012; Gajda et al., 2017). The simplicity of the design is allowing to configure any number of units in parallel electrical configuration and use them in any given wastewater tank as a floating system. This includes MFC use in urinal tanks to power devices in remote locations or in large wastewater treatment plants to lower energy cost. This might also help to solve the electricity and sanitation problem in the Developing World. Efficient utilization and scale-up allows the technology to come out of the laboratory to field trails to become useful to society and the environment. MFCs for wastewater treatment might benefit from electrically independent and compartmentalized modules similar to chemical fuel cells, where MFC units are modular, having many electrochemical cells with short distances between anode and cathode. Here the distance is kept to the minimum having the ceramic membrane as the separator where the spacing would be the function of the ceramic thickness. For this test, the small terracotta cylinders are the thinnest separators used in this study and it might be one of the indications of its good performance as shown previously (Merino Jimenez et al., 2017).

### The Modular Boxes and the Stack

**FIGURE 5:**
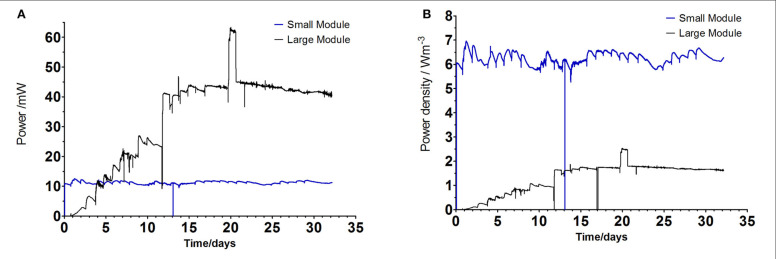
(A) Actual power (B) Power density of the tested miniaturized module in comparison to large scale module from previous study (Ieropoulos et al., 2016).

Due to ceramics availability and cost of the electrode material the SFFC 14 units were used to construct stack modules.**Figure 5** demonstrates the performance of one module made of 28 MFCs (SFFC 14 units shown in the individual study) in comparison to the large stack. While the absolute power obtained from the small module was over 4 times lower than large module, the power density increased up to 3 times (**Figure 5B**). It is worth mentioning that the large module was assembled using 36 large MFC units therefore the absolute power per MFC unit was 1.2 mW for the large and 0.4 mW for the small module which is in line with the individual MFC tests in **Figure 3**. Feeding for the small units required to be more frequent as it was supplied daily while the large module required less frequent replenishment. Therefore, the small scale modules would benefit from lower volume of processed anolyte and smaller footprint of the assembled system.

**FIGURE 6:**
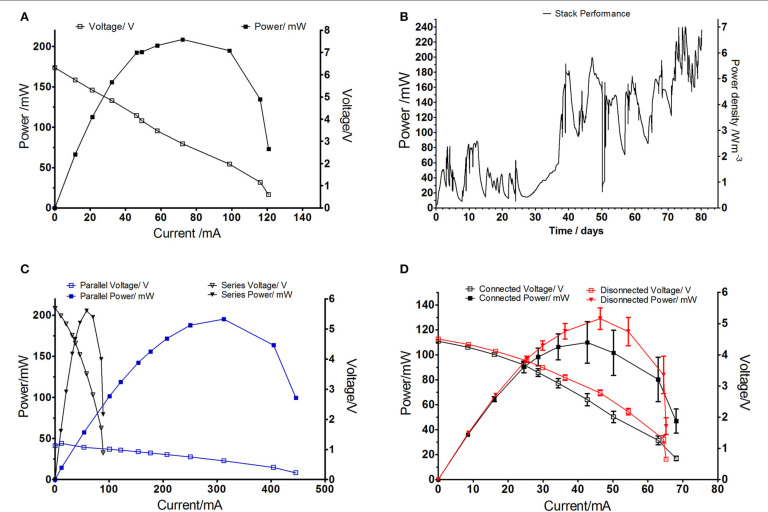
(A) Polarization experiment performed on the stack of 20 module boxes tested in laboratory conditions, (B) Power performance of the whole stack in parallel/series configuration, (C) Polarization curve experiments performed on parallel and series configuration of the paired module units, (D) Polarization curve

The modules were assembled in 20–module stack making a total of 560 MFCs as shown in **Figure 2**. Power performance was monitored over the period of 80 days and it shown up to 245 mW which corresponds to 6.8 W/m^3^ (**Figure 6B**), compared to its predecessor, large-scale stack with large terracotta units and the total of 300 L was producing 1.3 W/m^3^ which suggests up to 5 fold improvement (Ieropoulos et al., 2016). The MFCs in the modules were electrically connected in parallel due to being suspended in close proximity in shared electrolyte. The modules were then connected in series and parallel configuration (two modules paired in parallel connection and resulting 10 pairs were connected in series) to show flexibility of the stack electrical configuration and stability in power output (**Figures 6A,B**). This combination was chosen to balance the number of MFC units connected in parallel (56 MFCs across paired modules) and in series (10 modular pairs) connections in order to minimize the possibility of voltage imbalances and avoid cell reversals (Aelterman et al., 2006) as both tested arrangements resulted in the same total power output (**Figure 6C**). The electrical reconfiguring of MFCs is important factor to increase charging efficiency of the peripheral components such as capacitors for the operation of practical applications (Papaharalabos et al., 2014). Fluidical connections showed the higher power output when the air gaps were introduced (**Figure 6D**) which indicates the need of liquid isolations between the modules that are to be connected in series due to parasitic current related to cross-conduction between the anodes (Ledezma et al., 2013).

**FIGURE 7:**
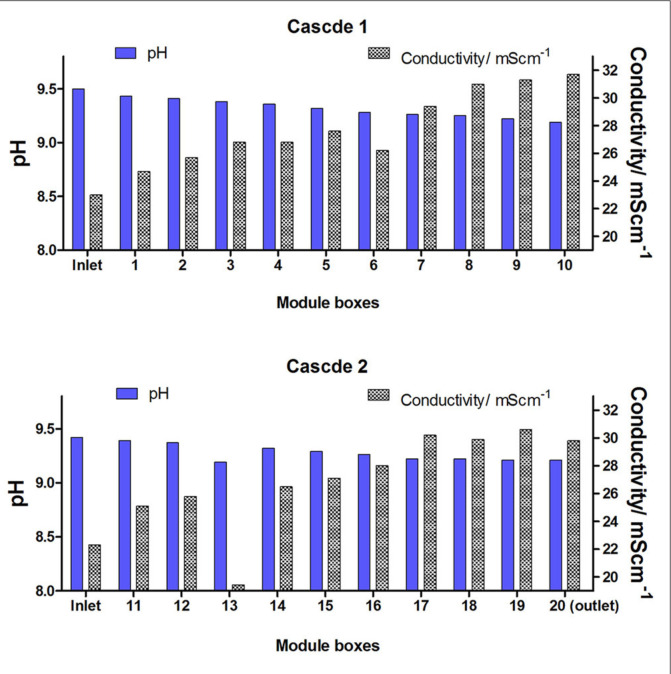
Physico-chemical properties of the two cascades in the stack in terms of the anolyte pH and conductivity.experiments performed on modules fluidically connected and disconnected with an air gap between the modules.

The pH and conductivity behavior (**Figure 7**) is similar to previously reported large stack operated in Glastonbury Festival in 2015 and showed anodic pH decrease in the cascade and conductivity increase (Ieropoulos et al., 2016). This might be due to the power and catholyte generation, biomass accumulation and evaporation losses. Both anode and the cathode were closely spaced therefore the durable electrical connection is one of the key challenges. Reducing internal reactor resistance and increasing cathodic reaction efficiency is the key challenge to maximize power. Hardware and operational constraints such as corrosion, electrical insulation rather than microbial activity, primarily contribute to limitations in MFC power and long term stability. While the modular construction of miniaturized fuel cells assemblies will allow the improvement in power generation of each module, increasing the number of modules will be affecting the mechanical and electrical properties. The challenges that remain in this technology include the electrical connection stability and avoiding corrosion, it is especially important in the small scale reactors where the available connection spacing is limited. The choice of the ceramic material used as a separator, its thickness, porosity, and composition is another area that requires further research as well as its availability and cost for mass manufacturing. While the COD and Total Nitrogen measurement did not reach desirable levels (**Figures S1**, **S2**) this is an area that requires further investigation in the future laboratory tests and field trials. This is also corresponding to the requirement for future characterization of the anodic population within a MFC operating on anaerobic sewage sludge and urine in order to study the metabolic pathways.

MFC architecture is inherently scalable due to good insulation between the electrodes and a compact architecture. Real implementations will also include the design of various ancillary components (wiring, tubing, cabling, insulation, connectors) that would be durable and adjusted to meet the electrical and fluidical configuration requirements. MFC scaled-up systems for real world implementations are complex in design and materials (Hiegemann et al., 2016) therefore simple and low maintenance systems would be favorable. Moreover, the catholyte producing MFC systems will allow long term operation without cathode clogging and production of disinfectant (Gajda et al., 2016). As the commercialization of of the components (in this case terracotta membrane) for MFCs will require mass manufacturing in a modular format manufacturing and economical approach into materials and mass (Logan et al., 2015) it is also limited by the availability production.

## CONCLUSIONS

This approach leads to improved power density generation from urine and the development of the off-the-grid electrochemical system that allows net generation of usable power and wastewater decontamination. Miniaturized scale ceramic based MFC modules inherently produce favorable conditions for high current/power generation because of (i) a large surface-to-volume ratio of ceramic membrane (ii) efficient mass transport due to smaller scale and (iii) short proton/electron travel distances. Also increased anode surface improves the output of the individual units and it is a factor that should be taken into account for further scale-up studies. The series and parallel electrical connectivity of stacked MFCs have been investigated as a mechanism to increase the overall voltage and current output and show a multi-module stack as a flexible tool to achieve desirable power and voltage levels required for peripheral hardware.

## SUPPLEMENTARY MATERIAL

The Supplementary Material for this article can be found online at: https://www.frontiersin.org/articles/10.3389/fenrg. 2018.00084/full#supplementary-material
