# Supplementary material for: Miniaturized Ceramic-Based Microbial Fuel Cell for Efficient Power Generation From Urine and Stack Development
Source: Front Energy Res. 2018 Oct 1;6:84. doi: 10.3389/fenrg.2018.00084 (PMC7705131; doi:10.3389/fenrg.2018.00084)
Supplement: Supplementary file 1 [file FER-06-084-s001.docx]

**Supplementary Information**

**Miniaturised ceramic –based Microbial Fuel Cell for efficient power generation from urine and stack development**

Iwona Gajda^a,*^, Andrew Stinchcombe^a^, Irene Merino Jimenez^a^, Grzegorz Pasternak^a^, Daniel Sanchez-Herranz ^a^, John Greenman^a,b^, Ioannis A. Ieropoulos^a,b*^

^a^ Bristol BioEnergy Centre, Bristol Robotics Laboratory, University of the West of England, BS16 1QY, UK

^b^ Department of Applied Sciences, University of the West of England, BS16 1QY, UK

^*^Corresponding authors: [Iwona.Gajda@uwe.ac.uk](mailto:Iwona.Gajda@uwe.ac.uk), [Ioannis.Ieropoulos@brl.ac.uk](mailto:Ioannis.Ieropoulos@brl.ac.uk)

Table S1. Composition of the artificial urine medium (AUM).

| **Component** | **Concentration (g/L)** | **Concentration (mmol/L)** |
| --- | --- | --- |
|  |  |  |
| Peptone | 10 |  |
| Yeast Extract | 5 |  |
| Urea | 5 | 85 |
| Sodium Chloride | 5.2 | 90 |
| Sodium Sulphate * 10 H2O | 3.2 | 10 |
| Potassium Dihydrogen Phosphate | 0.95 | 7 |
| Di-potassium Hydrogen Phosphate | 1.2 | 7 |

Figure S1. COD values measured in both MFC cascades. The numbers indicate module position in the stack where the module 10 is the outlet of each cascade.

Figure S2. Total Nitrogen (TN) values measured in both MFC cascades. The numbers indicate module position in the stack where the module 10 is the outlet of each cascade.
